# Optimization of the Diagnosis of Central Nervous System Infections in Vietnamese Hospitals: Results From a Retrospective Multicenter Study

**DOI:** 10.1093/ofid/ofae531

**Published:** 2024-09-13

**Authors:** Do Van Dong, Sébastien Boutin, Vu Viet Sang, Nguyen Dang Manh, Nghiem Xuan Hoan, Hoang Xuan Quang, Tran Thi Lien, Van Dinh Trang, Nguyen Trong The, Le Thi Kieu Linh, Kristina Schmauder, Viola Ueltzhöffer, Nourhane Hafza, Susanne Hauswaldt, Jan Rupp, Peter G Kremsner, Le Huu Song, Dennis Nurjadi, Silke Peter, Thirumalaisamy P Velavan

**Affiliations:** Institute of Tropical Medicine, Universitätsklinikum Tübingen, Tübingen, Germany; 108 Military Central Hospital, Hanoi, Vietnam; Vietnamese German Center for Medical Research (VG-CARE), Hanoi, Vietnam; Institute of Medical Microbiology and Clinic for Infectious Diseases, University of Lübeck and University Medical Center Schleswig-Holstein, Lübeck, Germany; German Center for Infection Research (DZIF), Partner Site Hamburg-Lübeck-Borstel-Riems, Lübeck, Germany; Airway Research Center North (ARCN), German Center for Lung Research (DZL), Lübeck, Germany; 108 Military Central Hospital, Hanoi, Vietnam; Vietnamese German Center for Medical Research (VG-CARE), Hanoi, Vietnam; 108 Military Central Hospital, Hanoi, Vietnam; Vietnamese German Center for Medical Research (VG-CARE), Hanoi, Vietnam; 108 Military Central Hospital, Hanoi, Vietnam; Vietnamese German Center for Medical Research (VG-CARE), Hanoi, Vietnam; 103 Military Hospital, Hanoi, Vietnam; Viet Tiep Friendship Hospital, Haiphong, Vietnam; Hai Phong University of Medicine and Pharmacy, Haiphong, Vietnam; National Hospital for Tropical Diseases, Hanoi, Vietnam; 108 Military Central Hospital, Hanoi, Vietnam; Vietnamese German Center for Medical Research (VG-CARE), Hanoi, Vietnam; Institute of Tropical Medicine, Universitätsklinikum Tübingen, Tübingen, Germany; Vietnamese German Center for Medical Research (VG-CARE), Hanoi, Vietnam; Institute of Medical Microbiology and Hygiene, Universitätsklinikum Tübingen, Tübingen, Germany; German Center for Infection Research (DZIF), Partner Site Tübingen, Tübingen, Germany; Institute of Medical Microbiology and Hygiene, Universitätsklinikum Tübingen, Tübingen, Germany; Institute of Tropical Medicine, Universitätsklinikum Tübingen, Tübingen, Germany; Institute of Medical Microbiology and Clinic for Infectious Diseases, University of Lübeck and University Medical Center Schleswig-Holstein, Lübeck, Germany; Institute of Medical Microbiology and Clinic for Infectious Diseases, University of Lübeck and University Medical Center Schleswig-Holstein, Lübeck, Germany; German Center for Infection Research (DZIF), Partner Site Hamburg-Lübeck-Borstel-Riems, Lübeck, Germany; Institute of Tropical Medicine, Universitätsklinikum Tübingen, Tübingen, Germany; Centre de Recherches Médicales de Lambaréné (CERMEL), Lambaréné, Gabon; 108 Military Central Hospital, Hanoi, Vietnam; Vietnamese German Center for Medical Research (VG-CARE), Hanoi, Vietnam; Institute of Medical Microbiology and Clinic for Infectious Diseases, University of Lübeck and University Medical Center Schleswig-Holstein, Lübeck, Germany; German Center for Infection Research (DZIF), Partner Site Hamburg-Lübeck-Borstel-Riems, Lübeck, Germany; Institute of Medical Microbiology and Hygiene, Universitätsklinikum Tübingen, Tübingen, Germany; German Center for Infection Research (DZIF), Partner Site Tübingen, Tübingen, Germany; Institute of Tropical Medicine, Universitätsklinikum Tübingen, Tübingen, Germany; Vietnamese German Center for Medical Research (VG-CARE), Hanoi, Vietnam; German Center for Infection Research (DZIF), Partner Site Tübingen, Tübingen, Germany; Faculty of Medicine, Duy Tan University, Danang, Vietnam

**Keywords:** cerebrospinal fluid (CSF), CNS infections, diagnostics, FAME, Vietnam

## Abstract

**Introduction:**

Central nervous system infections pose significant health challenges, particularly in low- and middle-income countries, because of high morbidity and mortality rates. Rapid and accurate diagnosis is essential for effective treatment to prevent adverse outcomes. Traditional culture-based diagnostics are often slow and lack specificity. This study evaluates the BioFire FilmArray Meningitis/Encephalitis (FAME) Panel against standard diagnostics in Vietnam to assess its clinical impact and suitability for local epidemiology.

**Methods:**

We conducted a prospective study involving 330 patients with suspected central nervous system infections at 4 hospitals in northern Vietnam from July 2022 to April 2023. Cerebrospinal fluid samples were analyzed using routine culture methods and FAME. We compared pathogen detection rates and assessed the potential clinical impact of FAME results on patient management.

**Results:**

Of the 330 cerebrospinal fluid specimens, 64 (19%) were positive by either conventional diagnostics (n = 48) and/or FAME (n = 33). The agreement between FAME and conventional diagnostics was 87%. Key pathogens *Mycobacterium tuberculosis* (n = 7), *Klebsiella pneumoniae* (n = 5), *Streptococcus suis* (n = 5), Epstein-Barr virus (n = 3), *Acinetobacter baumannii* (n = 1), and *Trichosporon asahii* (n = 1) were not detected by FAME. Classical meningitis parameter clinical symptoms, altered glucose, protein, and pleocytosis were good predictors of FAME positivity, indicating their utility in optimizing local diagnostic algorithms.

**Conclusions:**

FAME complements traditional diagnostics by offering rapid and broad pathogen detection, crucial for timely and appropriate therapy. However, its effectiveness varies with local epidemiology, and it should not replace conventional methods entirely. Tailoring diagnostic panels to regional pathogen prevalence is recommended to enhance diagnostic accuracy and clinical outcomes in low- and middle-income countries.

Central nervous system (CNS) infections represent 1 of the more severe infections associated with high morbidity and mortality, particularly in low- and middle-income countries (LMICs) [1]. Timely diagnosis and appropriate antibiotic therapy are crucial for effectively managing CNS infections because any delay in initiating such treatment is associated with unfavorable outcomes and potential sequelae [2]. Surviving individuals may experience substantial long-term consequences such as cognitive deficit, bilateral hearing loss, motor deficit, seizures, and visual impairment [3].

The diagnosis of CNS infections is challenging and typically involves the collection of cerebrospinal fluid (CSF) by lumbar puncture for culture-based microbiological diagnostics. Routine CSF diagnostics, including evaluation of cytological and biochemical parameters and clinical features, can provide insight into the etiology of the infection and attempt to distinguish between bacterial, viral, or fungal origins [4]. However, these parameters often lack the specificity to distinguish the infecting agent for informed decisions on appropriate empirical antimicrobial therapy. Consequently, this limitation often results in the widespread prescription of broad-spectrum antibiotics, contributing to selection pressure and the emergence of antibiotic resistance.

In addition, the traditional culture-based microbiological approach, suffers from time constraints and potential sensitivity issues, especially when patients have received prior antibiotic treatment [5]. Recognizing these limitations, molecular diagnostics have emerged as a valuable addition to the diagnostic toolkit because of their rapid turnaround time. Most commercially available molecular tests for the diagnosis of CNS infections are targeted polymerase chain reaction (PCR) assays designed to identify the most common pathogens. However, it is important to note that these tests often reflect general epidemiologic trends rather than being tailored to specific regional contexts, particularly in LMICs where epidemiologic patterns can differ significantly from those in high-income countries. In particular, there has been limited research in resource-limited settings, with only 1 study identified in Myanmar [6], whereas other studies in Asia have predominantly been conducted in non-LMIC settings, such as Taiwan [7] and Korea [8].

In this context, we evaluated the additional benefit of implementing the BioFire FilmArray Meningitis/Encephalitis Panel (FAME) compared with the standard-of-care diagnostics (culture and an in-house multiplex PCR panel) in 4 hospitals around Hanoi, northern Vietnam. The primary objective was to investigate whether the targets included in FAME were suitable for the local epidemiology in Vietnam; the secondary objective was to assess whether the implementation of such a panel would have had a meaningful clinical impact.

## METHODS

### Ethical Approval Statement

Written informed consent was obtained from all hospitalized patients and/or their relatives and from parents if subjects were age <18 years old. The study protocol was approved by the 108 Military Central Hospital (108 MCH) institutional review board (108MCH/RES/MENTNGITIS-V-D3-25042017). All experiments were conducted in accordance with International Council for Harmonization-Good Clinical Practice/Good Clinical Laboratory Practice (ICH-GCP/GCLP) guidelines and regulations.

#### Study Population and Study Design

Patients with suspected CNS infections with clinical signs of CNS infection were recruited prospectively in 4 hospitals in northern Vietnam, including 108 MCH, National Hospital for Tropical Diseases (NHTD), 103 Military Hospital (103 MH), Hanoi, and Viet Tiep Friendship Hospital (VT), Haiphong, Vietnam, between 1 July 2022 and 30 April 2023. Patients were eligible for the study if they had clinical signs of suspected CNS infections, based on the World Health Organization case definition modified by Dubot-Pérès et al [9]. Patient recruitment was at the discretion of the treating physician and followed local clinical practice. The inclusion criteria required patients to have a fever or an axillary temperature >37.5 °C and to exhibit a combination of at least 2 of the following symptoms: focal neurologic deficits, Glasgow Coma Scale abnormalities, seizures, neck stiffness, and signs of altered mental status. Exclusion criteria included: (1) patients with noninfectious, noninflammatory neurological disorders; patients not presenting to the emergency department; patients transferred from other hospitals; or those lacking an acute indication for a lumbar puncture in the emergency department; patients with contraindications to lumbar puncture, such as an intracranial space-occupying lesion with mass effect, a mass in the posterior fossa, abnormal intracranial pressure, or a local skin infection at the lumbar puncture site; (2) patients with an incomplete clinical history; and (3) patients who have not consented to the study.

A total of 330 CSF samples from 330 patients with suspected CNS infections were analyzed (199 samples collected on admission and 131 during hospitalization). Case record forms were used to collect data on demographics, medical history, clinical features on admission and subclinical findings on admission or during hospitalization, clinical course, treatment, outcome, and neurological findings on discharge. Patients were diagnosed and treated according to the clinical algorithm and management of the respective hospital. Clinical outcome was defined according to the Glasgow Outcome Scale [10]. The CSF samples were collected at various hospital sites in Vietnam and stored at −80 °C. They were then transported to Germany, where all CSF samples were analyzed using the FAME assay. The cold chain was rigorously maintained throughout the entire process to ensure the samples integrity during transportation.

#### Standard-of-care Laboratory Diagnostics

Routine CSF tests included cell counts and differential counts, glucose and total protein analyses and CSF bacterial cultures. If required, additional tests such as CSF fungal culture and polymerase chain reaction (PCR) tests for specific bacteria, and viruses were also performed. The microbiological culture procedures were similar on all study sites. Briefly, 1 mL of CSF was collected from a lumbar puncture for microbiological culture. Cultures were performed using the BACTEC Plus Aerobic/F System (Becton–Dickinson, Franklin Lakes, NJ, USA) at 36 °C with CO_2_ for 18–72 hours. Each positive culture was grown on a selective medium such as blood agar, chocolate agar, and MacConkey agar (Merck, Kenilworth, NJ, USA). When bacterial growth was detected, colonies were selected for species identification using the matrix-assisted laser desorption/ionization time of flight VITEK MS system for automated microbial identification and antimicrobial susceptibility testing was performed using the automated VITEK®2 compact system (BioMérieux, Lyon, France). PCR is performed at the request of physicians, in addition to routine diagnostics. An overview of the coverage of the PCR panel at the different study sites is summarized in Supplementary Table 1.

#### BioFire FilmArray Meningitis/Encephalitis Assays

All frozen CSF samples collected from patients by lumbar puncture were analyzed with FAME following manufacturer's instructions. In brief, frozen, noncentrifuged CSF (200 µL) was placed in the bag after the hydration solution had been injected and processed according to the manufacturer's instructions. FAME panel included 14 pathogens, namely *Streptococcus pneumoniae*, *Neisseria meningitidis*, *Streptococcus agalactiae*, *Haemophilus influenzae*, *Listeria monocytogenes*, *Escherichia coli K1*, herpes simplex virus (HSV) 1 and HSV 2, human herpes virus 6, cytomegalovirus (CMV), Enterovirus, human parechovirus, varicella zoster virus (VZV), and *Cryptococcus neoformans/gattii*.

#### Data Analysis

Descriptive analysis was performed using SPSS Statistics 28 (IBM). The presence of pleocytosis, was quantitatively defined by 2 separate cutoff ranges for the corrected white blood cell count in CSF: ≥ 5 cells/mm^3^ and ≥10 cells/mm^3^ [11]. Abnormal CSF glucose and protein levels were defined for all patients as values of <2.8 mmol/L or >4.2 mmol/L and <0.10 g/L and >0.25 g/L, respectively, based on the 108 MCH criteria. Discordant results were defined as divergent results only when the pathogen was included in the standard of care diagnostic panel. The association of the clinical parameters with the positivity of detection by conventional or FAME method was evaluated using a Random Forest model using the positive/negative status as a prediction of the importance (Gini) of all available clinical parameters as predictors using the package Random Forest in R 4.3.3. Missing values were handled using the command na.roughfix, which replaced the quantitative missing value with the overall population medians and the qualitative values with the most frequent values in the population.

## RESULTS

### General Characteristics of the Study Cohort

A total of 330 patients from 456 patients with suspected CNS infections (111 fulfilling exclusion criteria and 17 nonconsent) were recruited in various hospitals around Hanoi, Vietnam (Supplementary Figure 1). Recruitment numbers varied by site: 40 patients from 103 MH, 52 from 108 MH, 136 from the NHTD, and 102 from VT Hospital. At 108 MH, 52 of 81 eligible patients were recruited, whereas the remaining 29 were excluded because of reasons such as transfer from other hospitals (n = 6 patients), incomplete clinical histories (n = 6), death (n = 2), not presenting directly to the emergency department (n = 4), lack of consent (n = 5), and family decisions to take elderly patients home (n = 6). At NHTD, 136 of 175 patients were recruited, with 6 declining consents and 33 fulfilling the exclusion criteria. At 103 MH, 40 of 60 patients were recruited, with 2 not consenting and 18 fulfilling the exclusion criteria. No further data were available from these 2 hospitals. At VT, 102 of 140 patients were recruited, with the remaining 38 patients excluded because of contraindications to lumbar puncture (n = 5), transfers from other hospitals (n = 13), lack of consent (n = 4), diagnosis was uncertain and inconclusive (n = 7), and no retrievable clinical history (n = 9). The median age of the cohort was 54 years (range, 11–97 years). Of these, 225 (68%) were male. The main clinical characteristics are summarized in Table 1. Regarding preexisting medical conditions, 27% of patients had hypertension, 19% had diabetes, and 6% had cardiac disease. Common clinical presentations included fever (83%), headache (67%), neck stiffness (63%), and altered mental status (defined by a Glasgow Coma Scale score below 14) in 43% of cases. Notably, 83% of patients had at least 2 of the following symptoms: headache, fever, neck stiffness, and altered mental status.

**Table 1.** Demographic and Clinical Characteristics of Vietnamese Patients With Central Nervous System Infections

General Characteristics	CNS Infections (n = 330)	Routine Positive Specimens (n = 48)	Routine Negative Specimens (n = 282)	*P* Value	FAME-positive Specimens (n = 33)	FAME-negative Specimens (n = 297)	*P* Value
Demographics							
Age (mean ± SD)	54 ± 19	53 ± 18	55 ± 19	.47	53 ± 18	55 ± 19	.62
Male sex—no. (%)	225 (68)	37 (77)	188 (67)	.15	24 (73)	201 (68)	.56
Underlying condition—no. (%)							
Hypertension	90 (27)	8 (17)	82 (29)	.07	10 (30)	80 (27)	.68
Diabetes	62 (19)	2 (4)	60 (21)	.005	4 (12)	58 (20)	.30
Cardiac disease	19 (6)	1 (2)	18 (6)	.33^a^	0 (0)	19 (6)	.24^a^
Alcoholism	17 (5)	8 (17)	9 (3)	<.001^a^	2 (6)	15 (5)	.68^a^
Chronic liver disease	18 (6)	4 (8)	14 (5)	.31^a^	1 (3)	17 (6)	1^a^
Chronic lung disease	17 (5)	3 (6)	14 (5)	.72^a^	3 (9)	14 (5)	.24^a^
Kidney disease	15 (5)	2 (4)	13 (5)	1^a^	1 (3)	14 (5)	1^a^
Immunosuppressive drugs	13 (4)	1 (2)	12 (4)	.7^a^	1 (3)	12 (4)	1^a^
Cancer	13 (4)	0 (0)	13 (5)	.23^a^	0 (0)	13 (4)	.38^a^
HIV-positive	9 (3)	1 (2)	8 (3)	1^a^	0 (0)	9 (3)	.61^a^
Risk factor**—**no. (%)							
Post neurosurgery	29 (9)	5 (10)	24 (9)	.59^a^	3 (9)	26 (9)	1^a^
Head trauma	31 (9)	2 (4)	29 (10)	.28^a^	3 (9)	28 (9)	1^a^
CSF shunt	5 (2)	0 (0)	5 (2)	1^a^	0 (0)	5 (2)	1^a^
Clinical features—no. (%)							
Fever (>37.5 °C)	273 (83)	46 (96)	227 (81)	.009	32 (97)	241 (81)	.02
Headache	221 (67)	37 (77)	184 (65)	.11	27 (82)	194 (65)	.06
Neck stiffness	207 (63)	40 (83)	167 (59)	.001	27 (82)	180 (61)	.02
Nausea/vomiting	99 (30)	21 (44)	78 (8)	.025	13 (39)	86 (29)	.21
Seizure	29 (9)	3 (6)	26 (9)	.78^a^	1 (3)	28 (9)	.34^a^
Focal neurologic deficits	20 (6)	4 (8)	16 (6)	.51^a^	2 (6)	18 (6)	1^a^
Glasgow Coma Score	13 ± 2	12 ± 2	13 ± 2	<.001	13 ± 2	13 ± 2	.62
< 14 (indicating altered mental status)—no. (%)	142 (43)	36 (75)	106 (38)	<.001	19 (58)	123 (41)	.08
At least 2 of 4 symptoms (headache, fever, stiff neck, and altered mental status)	273 (83)	45 (94)	228 (81)	.03	32 (97)	241 (81)	.02
Outcome							
Death	32 (10)	4 (8)	28 (10)		3 (9)	29 (10)	
Vegetative state	29 (9)	10 (21)	19 (7)		3 (9)	26 (9)	
Severe disability	64 (19)	8 (17)	56 (20)		4 (12)	60 (20)	
Moderate disability	62 (19)	5 (10)	57 (20)		5 (15)	57 (19)	
Mild or no disability	143 (43)	21 (44)	122 (43)		18 (55)	125 (42)	
Cerebrospinal fluid							
Cell count (cells/mm^3^)^b^							
mean ± SD	1032 ± 5897	4898 ± 14437	376 ± 1726	<.001	3315 ± 7751	761 ± 5601	.007
Pleocytosis of 5 cells/mm^3^	225 (69)	42 (89)	183 (66)	.001	29 (88)	196 (67)	.015
Pleocytosis of 10 cells/mm^3^	175 (54)	39 (83)	136 (49)	<.001	26 (79)	149 (51)	.003
Protein (g/L)^c^							
Mean ± SD	1.56 ± 2.78	3.54 ± 4.01	1.22 ± 2.36	<.001	2.38 ± 2.36	1.47 ± 2.82	.07
Abnormal protein	307 (94)	48 (100)	259 (93)	.089^a^	33 (100)	274 (94)	.24^a^
Glucose (mmol/L)^c^							
Mean ± SD	4.16 ± 2.96	2.11 ± 1.85	4.50 ± 2.98	<.001	3.35 ± 3.85	4.25 ± 2.84	.09
Abnormal glucose	188 (58)	36 (77)	152 (54)	.005	23 (70)	165 (56)	.10

Abbreviation: CSF, cerebrospinal fluid; FAME, BioFire FilmArray Meningitis/Encephalitis.

^a^Fisher exact test.

^b^The CSF leukocyte count was determined in 324 patients; CSF specimens from 6 patients had too many leukocytes for an exact count to be performed (missing values, n = 6 for FAME negative, n = 1 for routine test positive and n = 5 for routine test negative).

^c^The protein and glucose levels in the CSF were determined in 326 patients; the remaining 4 CSF samples were not measured (missing values, n = 4 for FAME negative and n = 4 for routine test negative).

### Detected Pathogens by FAME and Conventional Diagnostics

**Figure 1.**
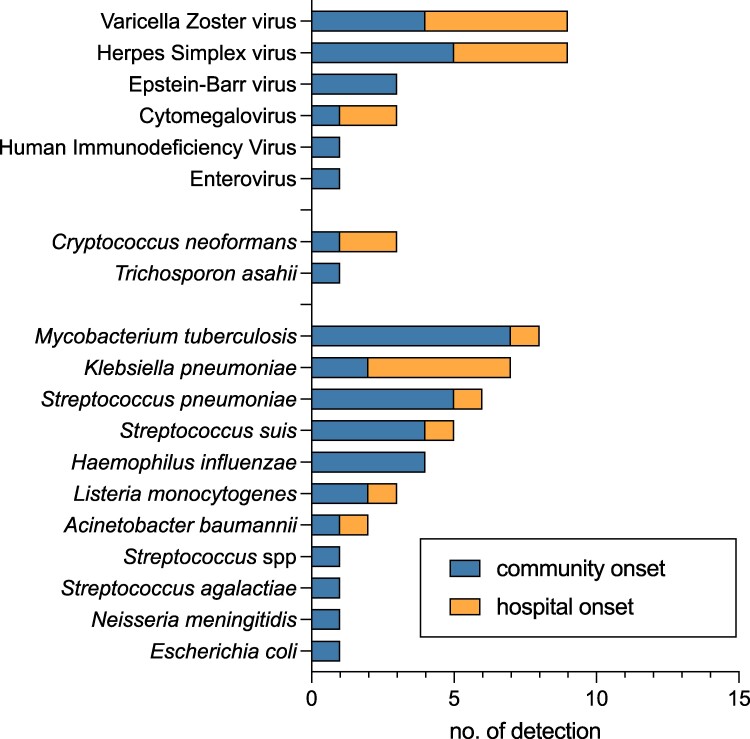
Distribution of bacterial, viral and fungal pathogens by onset of infection.

In total, 64 of 330 (19%) samples yielded positive results for bacterial, fungal, and/or viral pathogens. In our study, tuberculous meningitis was the leading cause of community-acquired CNS infections with community onset in 7 of 8 positive cases. The leading causes of community-acquired bacterial CNS infections were *S pneumoniae* with community-onset in 5 of 6, *Streptococcus suis* with 4 of 5, and *H influenzae* with 4 of 5 cases. Meanwhile, *Klebsiella pneumoniae* with hospital-onset in 5 of 7 positive cases, is the most common bacterial pathogen detected in hospital-acquired CNS infections. Among viral pathogens, VZV and HSV were predominant and equally represented in community-onset and hospital-onset viral CNS infections. *C neoformans* was the most common fungal pathogen but was only sporadically detected. *E coli,* HSV-2, human herpes virus 6 and human parechovirus were not detected in the study with FAME (Figure 1).

**Figure 2.**
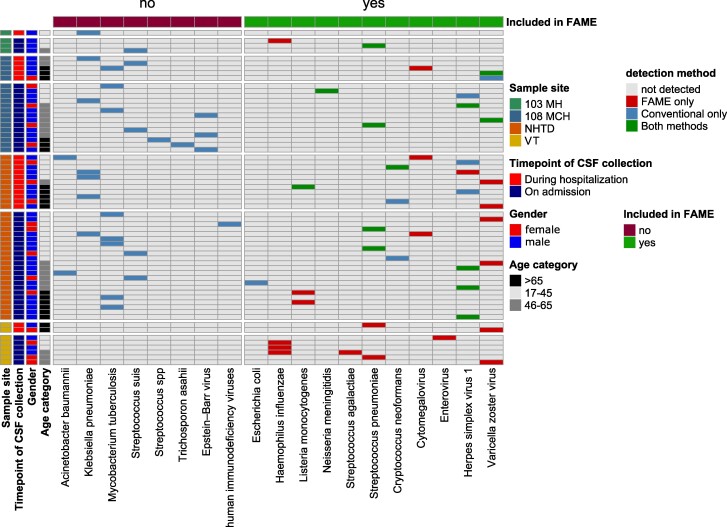
Overview of pathogen detection by various diagnostic algorithms. 103 MH, 103 Military Hospital; 108 MCH, 108 Military Central Hospital; NHTD, National Hospital for Tropical Diseases; VT, Viet Tiep Friendship Hospital; FAME, BioFire FilmArray Meningitis/Encephalitis Panel; CSF, cerebrospinal fluid.

The laboratory procedures for culture-based diagnostics were consistent across all 4 study sites. However, there were variations in the range of pathogens covered by the local molecular diagnostics (PCR) panels (Supplementary Table 1). Consequently, the evaluation of the results of the FAME and the conventional diagnostics results (culture plus molecular method) differed between the centers (Figure 2). Overall, in 48 CSF samples, pathogens were detected by conventional method and in 33 samples, a positive result was obtained in the FAME.

The positivity rates at the different study sites were 10% (4/40) for 103 MH, 37% (19/52) for 108 MCH, 24% (33/136) for NHTD, and 8% (8/102) for VT, detected by either conventional methods or FAME. As expected, the highest concordance was observed at 108 MCH, which had the most extensive PCR panel, with 63% (5/8 pathogens) concordance in pathogen detection between conventional diagnostics and FAME. Conversely, the lowest concordance was observed at VT, the center without molecular diagnostics. At the study site VT, of the 8 pathogens covered by FAME, none was detected by conventional diagnostics.

After a thorough review of patient data and charts of discrepancies between FAME results and conventional diagnostic methods, there were 286 matched sample results with an overall match rate of 87% (286/330). In 6 CSF specimens, FAME yielded positive results, primarily for the detection of viral pathogens (3 CMV and 1 HSV-1), as well as for *H influenzae* (n = 2) (Table 2). For the samples with positive CMV and HSV-1 signals in FAME, another pathogen (*K pneumoniae*, *A baumannii*, and *M tuberculosis*) could be detected by blood culture or other molecular methods, which may better explain the clinical presentation. However, no specific PCR was ordered for the remaining two samples with *H influenzae* detection by FAME, so we cannot be sure whether these samples were false positives. In addition, there were 4 pathogens, *E coli* (1/1), *C neoformans* (2/3), HSV-1 (3/8), and VZV (1/9), that were included in the FAME but yielded negative results (Supplementary Tables 2 and 3). Although *M tuberculosis*, *K pneumoniae*, and *S suis* were among the most detected bacterial pathogens, these targets were not included in the FAME panel.

**Table 2.** Comparison of FAME and Comparator Test Results for the Diagnosis of CNS Infections in Vietnam

Results Interpretation	FAME	Comparator Methods (CSF Culture and/or PCR)	Clinical Diagnosis	No. of Patients	Identified Pathogens
Concordant results					
	Positive	Positive (concordant pathogen)	Agreement	13	*S pneumoniae* (n = 4), HSV-1 (n = 4), VZV (n = 2)*, N meningitidis* (n = 1), *L monocytogenes* (n = 1), *C neoformans* (n = 1).
	Positive	Negative	Agreement	7	*L monocytogenes* (n = 3), *S pneumoniae* (n = 2), *H influenzae* (n = 1), *H influenzae + S agalactiae* (n = 1)
	Negative	Negative	-	266	*…*
Discordant results					
Expected pathogen was included in the FAME panel					
	Negative	Positive (pathogens included in FAME)	Disagreement	7	HSV-1 (n = 3), *C neoformans* (n = 2), *E coli* (n = 1), VZV (n = 1)
	Positive	Negative	Disagreement	2	*H influenzae* (n = 2)
	Positive	Positive (discordant pathogen)	Disagreement	4	CMV/*M tuberculosis* (n = 1), HSV-1/*K pneumoniae* (n = 1), CMV/*A baumannii* (n = 1), CMV/*K pneumoniae* (n = 1)
Not included in the FAME panel or standard-of-care diagnostic panel					
	Positive	Not performed/not ordered	NA	7	VZV (n = 6) and Enterovirus (n = 1)
	Not included	Positive	NA	24	*M tuberculosis* (n = 7), *K pneumoniae* (n = 5), *S suis* (n = 5), Epstein–Barr virus (n = 3), *A baumannii* (n = 1), *Trichosporon asahii* (n = 1), *Streptococcus* spp (n = 1), and HIV (n = 1)

Abbreviations: CNS, central nervous system; FAME, BioFire FilmArray Meningitis/Encephalitis; HSV, herpes simplex virus; NA, not applicable; VZV, varicella zoster virus.

### Implications for Antimicrobial and Diagnostic Stewardship

The national recommendation for antibiotic treatment regimens for patients with suspected bacterial meningitis is summarized in Supplementary Table 4. The clinical data and the laboratory results were analyzed retrospectively to determine the potential impact on antibiotic prescribing in our study cohort. Of the 33 FAME-positive cases, 20/33 (61%) patients were indicated for inappropriate empirical antibiotic therapy. If the FAME results had been available earlier, 20 patients would have benefited from FAME diagnosis, including antibiotic discontinuation in 8 patients, antibiotic de-escalation in 5, antibiotic change in 3, optimization of therapy in 2, antibiotic de-escalation and antiviral drug discontinuation in 1, and antiviral drug discontinuation in 1 (Supplementary Table 2).

After extensive analysis by machine learning algorithms of various clinical parameters and patient data collected for this study to predict pathogen positivity using either conventional methods or FAME indicated that conventional diagnostic markers emerge as the most important predictors (error rate of 10.61% for FAME and 11.52% for routine diagnostics). Specifically, these markers include CSF cell counts, abnormal (low) glucose levels, and elevated protein levels. Consequently, the integration of complementary and broadly targeted molecular diagnostics for CNS infections has the potential to improve diagnostic accuracy in patients with suspected CNS infections who present with these clinical parameters (Supplementary Figure 2).

## DISCUSSION

Our study showed that targeted molecular diagnostics, such as FAME or in-house molecular diagnostics, can complement conventional culture-based microbiological diagnostics in detecting CNS infections. Furthermore, clinical presentation and laboratory parameters such as headache, neck stiffness, and elevated CSF cell count, protein, and glucose levels can be used to improve the diagnostic algorithm for CNS infections in Vietnam. Implementing an evidence-based diagnostic algorithm in the sense of combined antimicrobial/diagnostic stewardship could increase cost-effectiveness by avoiding overtesting or overordering of expensive molecular tests [11, 12]. A study by Broadhurst et al [13]. showed that the positivity rate of FAME could be increased from 11.5% (53/459) to 18.6% (49/263) by implementing a testing algorithm.

Inappropriate administration and/or overuse of antibiotics to patients is one of the main causes of the emergence of resistant bacteria, leading to significantly longer hospital stays and considerable costs for the healthcare system as well as for patients and their families [14]. The implementation of rapid molecular diagnostics, such as FAME, could reduce the turnaround time of microbiological diagnostics of CSF, potentially leading to faster optimization/adjustment of antimicrobial therapy (Supplementary Table 2). Thus, complementing FAME to antimicrobial stewardship programme interventions may help to enhance clinical impact avoiding the overuse of antimicrobial substances.

Another benefit of implementing molecular diagnostics for diagnosing CSF infection is the possibility of simultaneous detection of bacterial, viral, and fungal pathogens. Moreover, molecular diagnostics have been shown to have a higher sensitivity for patients previously treated with antibiotics before sampling [15]. Diagnostic results should always be interpreted in conjunction with the clinical features, CSF analysis, and other available microbiological results. In our study, FAME contributed to the identification of more than 9 bacteria in culture-negative CSF samples, including *L monocytogenes* (n = 3), *H influenzae* (n = 3), *S pneumoniae* (n = 2), and 1 case of co-detection (*H influenzae* and *S agalactiae*), of which 4 patients had taken antibiotics before lumbar puncture. Potential explanations for the discordance in the detection of *H influenzae* and *S pneumoniae* could the higher sensitivity of molecular methods than conventional culture [5] or false positive signals of the FAME assay, which has been reported previously [16].

However, we also observed several false-positive and false-negative signals by FAME in this study. After a thorough review of the patient data and charts of the inconsistencies between the results obtained by FAME and conventional diagnostic methods, there were 7 instances in which FAME produced false-negative results for pathogens included in its panel. These included HSV-1 (n = 3), *C neoformans* (n = 2), *E coli* (n = 1), and VZV (n = 1). Notably, patients in these cases had clinical symptoms consistent with a CNS infection, which were supported by the results of conventional diagnostic tests. It is of note that only *E coli* with a K1 capsule type is included in the FAME PCR panel; non-K1 *E coli* strains will therefore not be detected. Although, *C neoformans* can be detected by the FAME, implementation of *Cryptococcus* antigen testing in addition to microscopy and culture remains an important strategy in the diagnosis and management of cryptococcal disease [17]. In 6 CSF samples, FAME yielded positive results in contrast to the standard-of-care diagnostics, primarily for the detection of viral pathogens (3 CMV and 1 HSV-1), as well as for *H influenzae* (n = 2), primarily for the detection of viral pathogens (3 CMV and 1 HSV-1), as well as for *H influenzae* (n = 2). It is noteworthy that for CMV and HSV-1, microbiologic analysis of CSF and/or blood cultures also revealed positive results for other bacterial pathogens (CMV/*M tuberculosis* n = 1, HSV/*K pneumoniae* n = 1, CMV/*A baumannii* n = 1, and CMV/*K pneumoniae* n = 1), which may provide a more accurate explanation for the observed symptoms. Two cases of *H influenzae* detection were likely to be false-positive signals because CSF analysis showed no pleocytosis, normal protein and glucose levels, and no agreement with the diagnosis at discharge (Supplementary Table 1). The determination of false-positive rates for *H influenzae* in CSF with FAME has been described previously by Zanella et al [18], in which only 1/17 FAME-positive samples could be confirmed by culture.

Our study highlights the importance of considering local epidemiology when selecting the most appropriate test panel for targeted diagnostics. FAME covers relevant pathogens causing community-acquired CNS infections but does not detect ESKAPE pathogens (*E faecium*, *S aureus*, *K pneumoniae*, *A baumannii*, *P aeruginosa,* and *Enterobacter spp*) that are considered to cause CNS infections in healthcare settings [12, 19]. Importantly, the performance of the panel is expected to depend on the epidemiology of CNS infections in different geographic regions [7]. In Vietnam, CNS infections are complex because they can be caused by a different pathogen spectrum compared to those in high-income and industrialized countries. *M tuberculosis,* which was also not included in the panel, remains a major public health challenge in Vietnam, where the incidence ranges from 260 to 399 per 100 000 population [20]. Meanwhile, *S suis* is one of the most common pathogens causing CNS infections in Vietnam and is associated with the consumption of undercooked pork or raw pig blood [21, 22]. In line with previous studies, our data suggest a lower sensitivity of the FAME assay in detecting HSV-1 and *C neoformans* [23–25] (Supplementary Table 3). Our results suggest that the implementation of a local epidemiology-adapted molecular diagnostic panel may be a better option than a commercial molecular diagnostic platform for resource-limited settings, as demonstrated by the performance of the standard of care diagnostics of the 108 MCH in this study.

In conclusion, our study suggests that the algorithm for effective use of FAME should be applied to patients with acute CNS infections not related to neurosurgery and a CSF pleocytosis of 5 cells/mm^3^. Although FAME is proving invaluable in the rapid detection of common meningitis pathogens, it should be used as a complementary rather than a replacement for conventional testing because it may not detect all pathogens associated with CNS infections. The implementation of FAME in a resource-limited laboratory setting with limited access to molecular methods could improve the diagnostic accuracy. Practitioners must exercise caution in interpreting and selecting results, considering the regional specificity of commercially available targeted molecular diagnostics.

## Supplementary Data

Supplementary materials are available at *Open Forum Infectious Diseases* online. Consisting of data provided by the authors to benefit the reader, the posted materials are not copyedited and are the sole responsibility of the authors, so questions or comments should be addressed to the corresponding author.

## Supplementary Material

ofae531_Supplementary_Data

## Notes

***Author Contributions.*** T.P.V. designed, supervised the study, and contributed to the study materials and assays. D.N. and S.P. were involved in the conceptualization and contributed to the study materials. D.V.D., L.T.L.K., K.S., and V.U. performed the experimental procedures. D.V.D., D.N., S.H., J.R., and S.B. were involved in the statistical analysis and validation of the results. D.V.D., V.V.S., N.D.M., N.X.H., H.X.Q., T.T.L., V.D.T., L.H.S., and N.T.T recruited the patients and contributed to the investigation materials for sampling procedures. D.V.D. wrote the first draft. T.P.V., D.N., S.H., J.R., P.K., D.V.D., and N.H. reviewed the first draft. All authors have read and approved the manuscript.

***Data availability.*** Not Applicable. The authors confirm that the data supporting the findings of this study are available within the article and in its supplementary material.

***Financial support*.** This study was funded by the PAN ASEAN Coalition for Epidemic and Outbreak Preparedness (PACE-UP; German Academic Exchange Service (DAAD) Project ID: 57592343). The funder has no role in the study design, data collection and analysis, decision to publish or preparation of the manuscript.

***Ethical approval and consent to participate.*** Written informed consent was obtained from all hospitalized patients and/or their relatives, and from parents if subjects were aged <18 years. The study protocol was approved by the 108 MCH institutional review board (108MCH/RES/MENTNGITIS-V-D3-25042017). All experiments were conducted in accordance with ICH GCP guidelines and regulations.

***Consent for publication***. All authors read and consented to publish the article.

## References

[1] GBD 2016 Meningitis Collaborators . Global, regional, and national burden of meningitis, 1990–2016: a systematic analysis for the global burden of disease study 2016. Lancet Neurol 2018; 17:1061–82. 30507391 10.1016/S1474-4422(18)30387-9 PMC6234314

[2] Bodilsen J , Dalager-Pedersen M, Schonheyder HC, Nielsen H. Time to antibiotic therapy and outcome in bacterial meningitis: a Danish population-based cohort study. BMC Infect Dis 2016; 16:392. 27507415 10.1186/s12879-016-1711-z PMC4977612

[3] Edmond K , Clark A, Korczak VS, Sanderson C, Griffiths UK, Rudan I. Global and regional risk of disabling sequelae from bacterial meningitis: a systematic review and meta-analysis. Lancet Infect Dis 2010; 10:317–28. 20417414 10.1016/S1473-3099(10)70048-7

[4] Spanos A , Harrell FE Jr, Durack DT. Differential diagnosis of acute meningitis. An analysis of the predictive value of initial observations. JAMA 1989; 262:2700–7. 2810603

[5] Brouwer MC , Tunkel AR, van de Beek D. Epidemiology, diagnosis, and antimicrobial treatment of acute bacterial meningitis. Clin Microbiol Rev 2010; 23:467–92. 20610819 10.1128/CMR.00070-09 PMC2901656

[6] Galardi MM , Sowa GM, Crockett CD, et al Pathogen and antibody identification in children with encephalitis in Myanmar. Ann Neurol 2023; 93:615–28. 36443898 10.1002/ana.26560

[7] Lee SH , Chen SY, Chien JY, Lee TF, Chen JM, Hsueh PR. Usefulness of the FilmArray meningitis/encephalitis (M/E) panel for the diagnosis of infectious meningitis and encephalitis in Taiwan. J Microbiol Immunol Infect 2019; 52:760–8. 31085115 10.1016/j.jmii.2019.04.005

[8] Roh S , Lee JH, Ha GY, et al Multicenter evaluation of the implementation Status of laboratory tests in Korea and the potential usefulness of a Multiplex PCR assay in patients with suspected central nervous system infections. Clin Lab 2020; 66. Available at: https://www.clin-lab-publications.com/article/3251 10.7754/Clin.Lab.2019.190420 32013348

[9] Dubot-Peres A , Mayxay M, Phetsouvanh R, et al Management of central nervous system infections, Vientiane, Laos, 2003–2011. Emerg Infect Dis 2019; 25:898–910. 31002063 10.3201/eid2505.180914 PMC6478220

[10] van de Beek D , de Gans J, Spanjaard L, Weisfelt M, Reitsma JB, Vermeulen M. Clinical features and prognostic factors in adults with bacterial meningitis. N Engl J Med 2004; 351:1849–59. 15509818 10.1056/NEJMoa040845

[11] Radmard S , Reid S, Ciryam P, et al Clinical utilization of the FilmArray meningitis/encephalitis (ME) Multiplex polymerase chain reaction (PCR) assay. Front Neurol 2019; 10:281. 30972012 10.3389/fneur.2019.00281 PMC6443843

[12] Tunkel AR , Hasbun R, Bhimraj A, et al 2017 Infectious Diseases Society of America's clinical practice guidelines for healthcare-associated ventriculitis and meningitis. Clin Infect Dis 2017; 64:e34–65. 28203777 10.1093/cid/ciw861 PMC5848239

[13] Broadhurst MJ , Dujari S, Budvytiene I, Pinsky BA, Gold CA, Banaei N. Utilization, yield, and accuracy of the FilmArray meningitis/encephalitis panel with diagnostic stewardship and testing algorithm. J Clin Microbiol 2020; 58:e00311-20. 32493787 10.1128/JCM.00311-20 PMC7448656

[14] Ventola CL . The antibiotic resistance crisis: part 1: causes and threats. P T 2015; 40:277–83. 25859123 PMC4378521

[15] Du B , Hua C, Xia Y, et al Evaluation of the BioFire FilmArray meningitis/encephalitis panel for the detection of bacteria and yeast in Chinese children. Ann Transl Med 2019; 7:437. 31700873 10.21037/atm.2019.08.103 PMC6803227

[16] Sunnerhagen T , Widen J, Handhal S, Ozkaya Sahin G. A retrospective observational study of 1000 consecutive patients tested with the FilmArray(R) meningitis/encephalitis panel: clinical diagnosis at discharge and microbiological findings. Sci Rep 2024; 14:4015. 38369552 10.1038/s41598-024-54621-9 PMC10874959

[17] Chang CC , Harrison TS, Bicanic TA, et al Global guideline for the diagnosis and management of cryptococcosis: an initiative of the ECMM and ISHAM in cooperation with the ASM. Lancet Infect Dis 2024; 24:e495–512. 38346436 10.1016/S1473-3099(23)00731-4 PMC11526416

[18] Zanella MC , Cherkaoui A, Hinic V, et al Unexpectedly high false-positive rates for Haemophilus influenzae using a meningoencephalitis syndromic PCR panel in two tertiary centers. Front Cell Infect Microbiol 2021; 11:639658. 33763388 10.3389/fcimb.2021.639658 PMC7982903

[19] Pallerla SR , Van Dong D, Linh LTK, et al Diagnosis of pathogens causing bacterial meningitis using nanopore sequencing in a resource-limited setting. Ann Clin Microbiol Antimicrob 2022; 21:39. 36064402 10.1186/s12941-022-00530-6 PMC9443622

[20] Nguyen HV , Tiemersma EW, Nguyen HB, et al The second national tuberculosis prevalence survey in Vietnam. PLoS One 2020; 15:e0236532. 32673361 10.1371/journal.pone.0236532 PMC7365399

[21] Nghia HD , Tu le TP, Wolbers M, et al Risk factors of Streptococcus suis infection in Vietnam. A case-control study. PLoS One 2011; 6:e17604. 21408132 10.1371/journal.pone.0017604 PMC3050921

[22] Wertheim HF , Nguyen HN, Taylor W, et al Streptococcus suis, an important cause of adult bacterial meningitis in northern Vietnam. PLoS One 2009; 4:e5973. 19543404 10.1371/journal.pone.0005973 PMC2696092

[23] Graf EH , Farquharson MV, Cardenas AM. Comparative evaluation of the FilmArray meningitis/encephalitis molecular panel in a pediatric population. Diagn Microbiol Infect Dis 2017; 87:92–4. 27771208 10.1016/j.diagmicrobio.2016.09.022

[24] Lindstrom J , Elfving K, Lindh M, Westin J, Studahl M. Assessment of the FilmArray ME panel in 4199 consecutively tested cerebrospinal fluid samples. Clin Microbiol Infect 2022; 28:79–84. 34015534 10.1016/j.cmi.2021.05.017

[25] Tansarli GS , Chapin KC. Diagnostic test accuracy of the BioFire(R) FilmArray(R) meningitis/encephalitis panel: a systematic review and meta-analysis. Clin Microbiol Infect 2020; 26:281–90. 31760115 10.1016/j.cmi.2019.11.016

