# Supplementary material for: Optimization of the Diagnosis of Central Nervous System Infections in Vietnamese Hospitals: Results From a Retrospective Multicenter Study
Source: Open Forum Infect Dis. 2024 Sep 13;11(9):ofae531. doi: 10.1093/ofid/ofae531 (PMC11429109; doi:10.1093/ofid/ofae531)
Supplement: ofae531_Supplementary_Data [file ofae531_supplementary_data.docx]

**Supplementary Figure S1. Overview of patient inclusion at four study sites.**

**
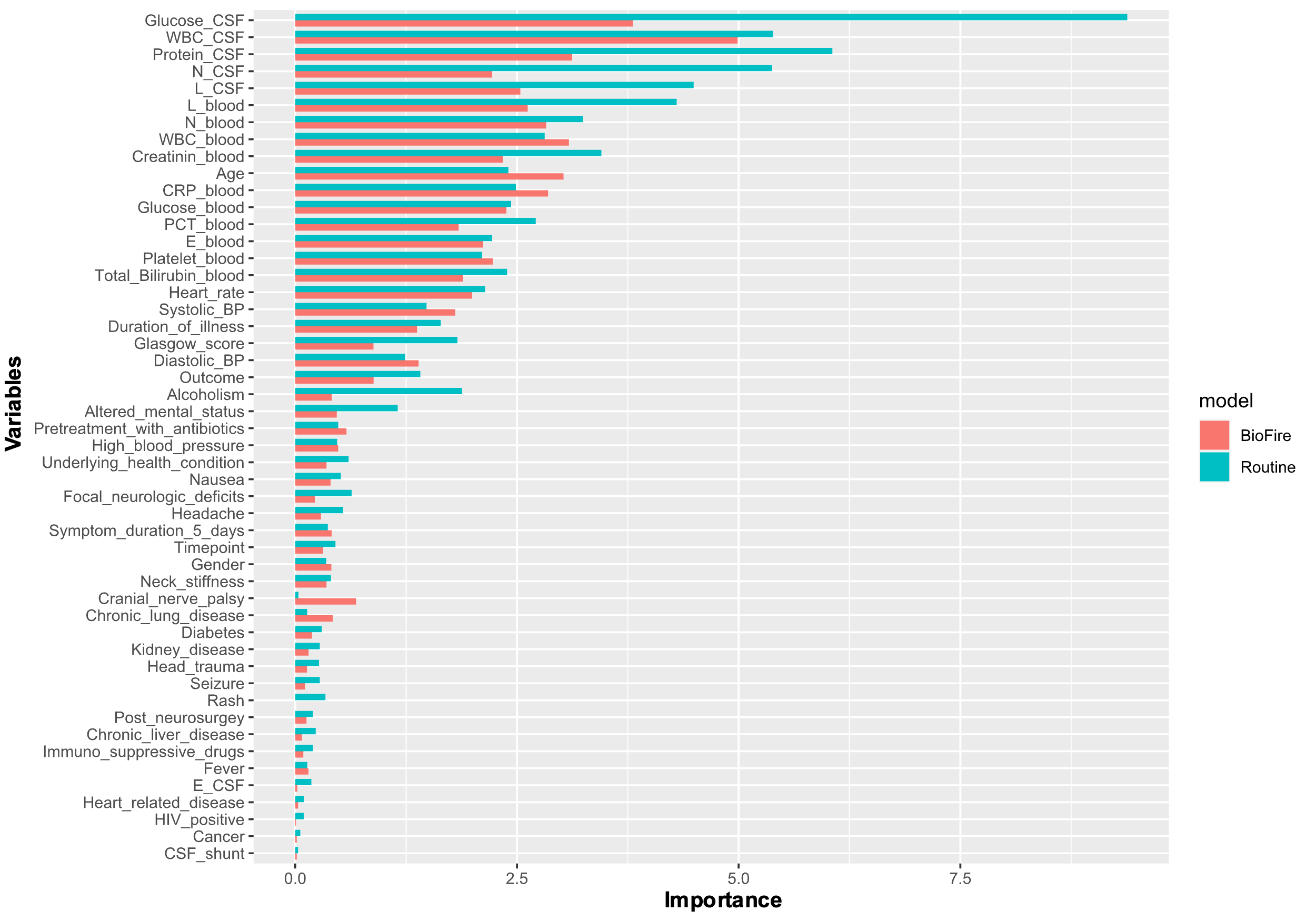
Supplementary Figure S2. Variable importance plot from both random forest regression model.**

| **FAME OOB estimate of  error rate: 10.61%** | | | |
| --- | --- | --- | --- |
| Confusion matrix | |  |  |
|  | **0** | **1** | **class.error** |
| **0** | 294 | 3 | 0.01010101 |
| **1** | 32 | 1 | 0.96969697 |
|  |  |  |  |
| **Routine Diagnostic OBB estimate of error rate: 11.52%** | | | |
| Confusion matrix |  |  |  |
|  | **0** | **1** | **class.error** |
| **0** | 278 | 4 | 0.0141844 |
| **1** | 34 | 14 | 0.7083333 |

Variable importance plot showing the relative importance of each clinical variables as a predictor of the positivity of detection of pathogen by either routine diagnostics or FAME.

**Abbreviations:** BP, blood pressure; CRP, C-reactive protein; CSF, cerebrospinal fluid; E, eosinophils; FAME, BioFire® FilmArray® Meningitis/Encephalitis Panel; HIV, human immunodeficiency viruses; L, lymphocyte; N, neutrophil; PCT, Procalcitonin; WBC, white blood cells.

**Supplementary Table S1: Standard-of-care diagnostics for diagnosing central nervous system infections in four Vietnamese hospitals.**

| **No.** | **Hospital** | **Routine diagnostics** | **Note** |
| --- | --- | --- | --- |
| 1 | 108 MCH | Bacteria: CSF Gram stain, CSF culture, blood culture, CSF realtime PCR, PCR-based assays and GenXpert. | Panel realtime PCR for detection of bacteria causing meningitis: *Streptococcus pneumoniae*, *Neisseria meningitidis*, *Streptococcus agalactiae*, *Haemophilus influenzae*, *Listeria monocytogenes*, *Escherichia coli K1.* |
|  |  |  | PCR-based assays: *Streptococcus suis, Streptococcus spp, Enterococcus spp, Staphylococcus spp, Klebsiella pneumoniae, Acinetobacter baumannii, Pseudomonas aeruginosa,* and *Mycobacterium tuberculosis.* |
|  |  |  | GenXpert: *M.tuberculosis.* |
|  |  | Viruses: CSF realtime PCR, PCR-based assays | Panel realtime PCR for detection of viruses: Herpes simplex virus 1/2, Cytomegalovirus, Epstein–Barr virus, Varicella zoster virus, Human herpes virus 6 and Human herpes virus 7. |
|  |  |  | PCR-based assays: Japanese encephalitis virus, Dengue virus, Enterovirus. |
|  |  | Fungi: CSF smear, fungal CSF culture |  |
| 2 | NHTD | Bacteria: CSF Gram stain, CSF culture, blood culture, CSF PCR-based assays and GenXpert. | PCR-based assays: *M.tuberculosis* |
|  |  |  | GenXpert: *M.tuberculosis* |
|  |  | Viruses: CSF PCR-based assays | PCR-based assays: Herpes simplex virus 1/2, *S.suis* |
|  |  | Fungi: CSF smear, fungal culture |  |
| 3 | VT | Bacteria: CSF Gram stain, CSF culture, blood culture |  |
|  |  | Fungi: CSF smear, fungal CSF culture |  |
| 4 | 103 MH | Bacteria: CSF Gram stain, CSF culture, CSF PCR-based assays and GenXpert, blood culture | PCR-based assays: *N.meningitis, M.tuberculosis* |
|  |  |  | GenXpert: *M.tuberculosis* |
|  |  | Viruses: CSF PCR-based assays | PCR-based assays: Cytomegalovirus, Epstein–Barr virus |
|  |  | Fungi: CSF smear, fungal CSF culture |  |

**Abbreviations**: CSF, Cerebrospinal fluid; PCR, Polymerase chain reaction; 108 MCH, 108 Military Central Hospital; 103 MH, 103 Military Hospital; NHTD, National Hospital for Tropical Diseases; VT, Viet Tiep Friendship Hospital.

Note: There are differences in the performance of four hospitals in routine diagnostics. 108 MCH, NHTD and 103 MH, the three national hospitals in Vietnam, are equipped with many modern devices, so the diagnostic capacity is better than VT, a provincial hospital.

| **Supplementary Table S2. Clinical and laboratory data of patients with CNS infections who were tested positive for FAME.** |
| --- |

| No. | Age/  Gender | Timepoint of CSF collection | FAME | CSF parameters | | | | Discrepancy investigation | | | | | Appropriate empirical therapy | Possible impact of FAME result on treatment | Interpretation^a^ | Outcome^b^ |
| --- | --- | --- | --- | --- | --- | --- | --- | --- | --- | --- | --- | --- | --- | --- | --- | --- |
|  |  |  |  | WBC | Pro | Glu | CSF culture | Antibiotic use before sampling | Empirical therapy | PCR/culture^a^ | Clinical diagnosis | Specific therapy |  |  |  |  |
| 1 | 59 yr/F | On admission | *S. pneumoniae* | 320 | 4.1 | 0.09 | *S. pneumoniae* | No | MER, AMK, DEX | CSF PCR and blood culture (+) *S. pneumoniae* | *S. pneumoniae* septic shock and meningitis | CRO, AMK, DEX | No | Antibiotic de-escalation | TP | 1 |
| 2 | 42 yr/M | On admission | *S. pneumoniae* | 18,050 | 6.7 | 0.01 | *S. pneumoniae* | No | CRO, DEX | Blood culture (+) *S. pneumoniae* | *S. pneumoniae* sepsis and meningitis | CRO, VAN, DEX | Yes | Optimisation of therapy | TP | 5 |
| 3 | 31 yr/F | On admission | *S. pneumoniae* | 1960 | 2.13 | 0.2 | *S. pneumoniae* | No | CRO, DEX | None | *S. pneumoniae* meningitis | MER | Yes | Optimisation of therapy | TP | 5 |
| 4 | 41 yr/M | On admission | *S. pneumoniae* | 10,460 | 1.65 | 0 | *S. pneumoniae* | No | CRO, LVF, MTP | Blood culture (+) *S. pneumoniae* | *S. pneumoniae* sepsis and meningitis | CRO, MTP | No | Antibiotic de-escalation | TP | 5 |
| 5 | 70 yr/M | During hospitalization | *S. pneumoniae* | 2062 | 2.69 | 5 | Negative | Yes | CRO, CIP, DEX | Blood culture (+) *S. pneumoniae* | *S. pneumoniae* sepsis and meningitis | CRO, CIP, DEX | No | Antibiotic de-escalation | TP | 5 |
| 6 | 48 yr/F | On admission | HSV-1 | 3 | 1.13 | 3.4 | Negative | No | CFT, ACV, DEX | CSF PCR (+) HSV-1 | HSV meningoencephalitis | ACV, DEX | No | Antibiotic discontinuation | TP | 5 |
| 7 | 79 yr/M | On admission | HSV-1 | 446 | 0.76 | 3.9 | Negative | No | ACV | CSF PCR (+) HSV-1 | HSV encephalitis | ACV | Yes | NA | TP | 3 |
| 8 | 63 yr/M | On admission | HSV-1 | 245 | 0.58 | 3.81 | Negative | Yes | ACV, MER, DEX | CSF PCR (+) HSV-1 | HSV encephalitis | ACV, DEX | No | Antibiotic discontinuation | TP | 4 |
| 9 | 58 yr/M | On admission | HSV-1 | 58 | 0.63 | 4.91 | Negative | Yes | ACV, CRO, DEX | CSF PCR (+) HSV-1 | HSV encephalitis | ACV, CRO, DEX | Yes | Antibiotic discontinuation | TP | 3 |
| 10 | 73 yr/M | During hospitalization | VZV | 6 | 0.77 | 3.3 | Negative | No | ACV | CSF PCR (+) VZV | VZV encephalitis | ACV, DEX | Yes | NA | TP | 3 |
| 11 | 58 yr/M | On admission | VZV | 1 | 0.8 | 5.1 | Negative | No | CRO, ACV, DEX | CSF PCR (+) VZV | VZV meningoencephalitis | ACV, DEX | No | Antibiotic discontinuation | TP | 5 |
| 12 | 20 yr/M | On admission | *N. meningitidis* | 8000 | 7.5 | 0 | *N. meningitidis* | Yes | MER, CIP, DEX | CSF PCR (+) *N. meningitidis* | *N. meningitidis* sepsis and meningitis | CRO, CIP | No | Antibiotic de-escalation | TP | 5 |
| 13 | 66 yr/M | During hospitalization | *L. monocytogenes* | 1624 | 3.53 | 0.68 | *L. monocytogenes* | Yes | MER, VAN | None | *L. monocytogenes* meningitis | MER, VAN, AMP | No | Antibiotic change | TP | 2 |
| 14 | 40 yr/M | During hospitalization | *C. neoformans* | 445 | 1.71 | 2.4 | Negative | Yes | MER, VAN | CSF fungal culture (+) *C. neoformans* | Cryptococcal meningitis | AMB, FLU | No | Antibiotic discontinuation | TP | 1 |
| 15 | 71 yr/M | During hospitalization | CMV | 54 | 1.31 | 1.7 | Negative | Yes | MER, VAN, AMK | CSF GeneXpert® (+) *M. tuberculosis* | Tuberculous meningitis | MER, AMK, LNZ, LVF, DEX | No | NA | FP^c^ | 1 |
| 16 | 44 yr/M | During hospitalization | HSV-1 | 9217 | 8.83 | 0.1 | *K. pneumoniae* | Yes | CRO, LVF, MET, DEX | Blood culture (+) *K. pneumoniae* | *K. pneumoniae* sepsis and meningitis | MER, AMK | No | NA | FP^c^ | 4 |
| 17 | 23 yr/M | During hospitalization | CMV | 38,731 | 6.34 | 0.1 | *A. baumannii* | Yes | MER, VAN, COL | None | *A. baumannii* meningitis, post neurosurgery | MER, COL | Yes | NA | FP^c^ | 2 |
| 18 | 37 yr/M | On admission | CMV | 199 | 6.09 | 4.33 | *K. pneumoniae* | Yes | MER, COL | None | *K. pneumoniae* meningitis and pneumoniae, post neurosurgery | MER, COL | Yes | NA | FP^c^ | 2 |
| 19 | 73 yr/M | On admission | *L. monocytogenes* | 828 | 0.94 | 1.9 | Negative | Yes | MER, AMP ACV | None | Bacterial meningitis | MER, AMP | No | Antibiotic de-escalation, antiviral drug discontinuation | TP | 3 |
| 20 | 69 yr/M | On admission | *L. monocytogenes* | 196 | 3.79 | 3.1 | Negative | Yes | MER, AMP | None | Bacterial meningitis | MER, AMP | No | Antibiotic de-escalation | TP | 5 |
| 21 | 66 yr/F | On admission | *L. monocytogenes* | 230 | 1.58 | 6.51 | Negative | Yes | MER, LNZ | None | Septic shock, bacterial meningitis | MER, LNZ | No | Antibiotic change | TP | 4 |
| 22 | 45 yr/M | On admission | *H. influenzae* | 2 | 0.36 | 4.1 | Negative | No | None | None | Vascular migraine | No specific treatment | NA | NA | FP^d^ | 5 |
| 23 | 40 yr/F | On admission | *H. influenzae* | 3 | 0.35 | 4.3 | Negative | No | CRO | None | Bacterial sepsis | CRO | Yes | NA | FP^d^ | 5 |
| 24 | 35 yr/M | On admission | *H. influenzae* | 4800 | 1.57 | 21.77 | Negative | No | MER | None | Bacterial meningitis | MER | No | Antibiotic change | TP | 5 |
| 25 | 49 yr/F | On admission | *S. pneumoniae* | 13,700 | 4.94 | 0.1 | Negative | No | CRO, DEX | None | Bacterial meningitis | CRO, DEX | Yes | NA | TP | 5 |
| 26 | 46 yr/M | On admission | *H. influenzae + S. agalactiae* | 9 | 0.87 | 3.1 | Negative | No | CRO, ACV, DEX | None | Bacterial meningitis | CRO, DEX | No | Antiviral drug discontinuation | TP | 4 |
| 27 | 25 yr/M | On admission | VZV | 10 | 0.36 | 3.94 | Negative | No | ACV | None | Shingles on the face, aseptic meningitis | ACV | Yes | NA | NA | 5 |
| 28 | 93 yr/M | During hospitalization | VZV | 92 | 2.53 | 2.68 | Negative | Yes | MER, ACV, LNZ | None | Viral meningoencephalitis and left external otitis | ACV, LNZ | No | Antibiotic discontinuation | NA | 4 |
| 29 | 55 yr/F | During hospitalization | VZV | 158 | 0.27 | 2.92 | Negative | Yes | CRO | CSF GeneXpert® (-) *M. tuberculosis* | Tuberculous meningitis | RHZ, MOX, DEX | No | NA | NA | 5 |
| 30 | 55 yr/M | On admission | VZV | 122 | 1.28 | 3.6 | Negative | Yes | RHZ, S, DEX | None | Tuberculous meningitis | RHZ, S, DEX | Yes | NA | NA | 5 |
| 31 | 50 yr/F | On admission | VZV | 66 | 0.62 | 2.4 | Negative | Yes | ACV | None | Viral meningoencephalitis | ACV | Yes | NA | NA | 5 |
| 32 | 83 yr/F | During hospitalization | VZV | 7 | 0.76 | 6.7 | Negative | Yes | CRO | None | Viral meningoencephalitis | CRO | No | Antibiotic discontinuation | NA | 5 |
| 33 | 36 yr/M | On admission | Enterovirus | 584 | 1.14 | 4.5 | Negative | No | CRO | None | Aseptic meningitis | CRO | No | Antibiotic discontinuation | NA | 5 |

**Abbreviations:** ACV, acyclovir; AMB, amphotericin B; AMK, amikacin; AMP, ampicillin; CFT, cefotaxime; CIP, ciprofloxacin; CMV, Cytomegalovirus, CNS, central nervous system; COL, colistin; CSF, cerebrospinal fluid; CRO, ceftriaxone; DEX, dexamethasone; F, female; FAME, BioFire® FilmArray® Meningitis/Encephalitis Panel; FLU, fluconazole; FP, false positive; Glu, glucose (mmol/L); HSV-1, herpes virus simplex 1; LNZ, linezolid; LVF, levofloxacin; M, male; MER, meropenem; MET, metronidazole; MOX, moxifloxacin; MTP, methylprednisolone; NA, Not applicable; Pro, protein (g/L); RHZ, R = rifampin; H = isoniazid; Z = pyrazinamide; E = ethambutol; S, streptomycin; TP, true positive; VAN, vancomycin; VZV, varicella zoster virus; WBC: white blood cells (cells/mm^3^).

^a^specific PCR was not ordered or performed

^b^Outcome: 1 death; 2: vegetative state; 3: severe disability; 4: moderate disability; 5: mild or no disability.

^c^false positive very likely due to the detection of other pathogens by culture or molecular methods

^d^false positive for the detection of *H. influenzae* possible (n=2). Confirmation by specific PCR was not performed.

**Supplementary Table S3. Clinical and laboratory data from patients with CNS infections positive by routine tests but negative by FAME.**

| No. | Age/Gender | Timepoint of CSF collection | Clinical diagnosis | WBC | Pro | Glu | CSF culture | Empirical therapy | Molecular method/culture | Specific therapy | Interpretation | Outcome^a^ |
| --- | --- | --- | --- | --- | --- | --- | --- | --- | --- | --- | --- | --- |
| 34 | 49 yr/M | On admission | Tuberculous meningitis | 1 | 1.17 | 3.5 | Negative | RHZE, DEX | CSF GeneXpert® (+) *M. tuberculosis* | RHZE, DEX | NA | 5 |
| 35 | 17 yr/F | On admission | Tuberculous meningitis | 297 | 4.06 | 2.3 | Negative | CRO, DEX | CSF GeneXpert® (+) *M. tuberculosis* | RHZE, DEX | NA | 5 |
| 36 | 68 yr/M | On admission | Tuberculous meningitis | 172 | 1.41 | 2.24 | Negative | MER, RHZ, MOX, DEX | CSF GeneXpert® (+) *M. tuberculosis* | MER, RHZ, S, DEX | NA | 3 |
| 37 | 38 yr/M | On admission | Tuberculous meningitis | 406 | 3.89 | 1.54 | Negative | CRO, LVF, RHZ, DEX | CSF GeneXpert® (+) *M. tuberculosis* | LVF, RHZ, DEX | NA | 5 |
| 38 | 41 yr/M | On admission | Tuberculous meningitis | 20 | 0.5 | 5.3 | Negative | LVF, LNZ, ZE | CSF GeneXpert® (+) *M. tuberculosis* | LVF, LNZ, ZE | NA | 4 |
| 39 | 72 yr/M | On admission | Tuberculous meningitis | 1096 | 3.36 | 0.8 | Negative | VAN, MER, DEX | CSF GeneXpert® (+) *M. tuberculosis* | MER, LNZ, LVF, RHZ, DEX | NA | 2 |
| 40 | 25 yr/M | On admission | Tuberculous meningitis | 136 | 6.1 | 1.08 | Negative | CRO, RHZ, S, DEX | CSF GeneXpert® (+) *M. tuberculosis* | RHZ, S, DEX | NA | 2 |
| 41 | 45 yr/M | On admission | *K. pneumoniae* meningitis and sepsis | 10,690 | 10.61 | 0 | *K. pneumoniae* | MER, COL, DEX | CSF PCR and blood culture (+) *K. pneumoniae* | MER, COL, DEX | NA | 5 |
| 42 | 44 yr/M | During hospitalization | *K. pneumoniae* meningitis | 3024 | 2.41 | 0.32 | *K. pneumoniae* | MER, LIN | None | MER, DEX | NA | 3 |
| 43 | 67 yr/M | During hospitalization | *K. pneumoniae* meningitis | 91,063 | 12.1 | 0.1 | *K. pneumoniae* | CRO, DEX | None | MER, AMK | NA | 4 |
| 44 | 18 yr/M | During hospitalization | *K. pneumoniae* meningitis | 3910 | 1.02 | 0.72 | *K. pneumoniae* | CFT | None | MER | NA | 2 |
| 45 | 55 yr/M | During hospitalization | *K. pneumoniae* meningitis and sepsis | 9 | 1.71 | 4.9 | Negative | MER, AMK | CSF PCR (+) *K. pneumoniae* | MER, AMK, COL | NA | 4 |
| 46 | 63 yr/M | During hospitalization | *S. suis* meningitis and sepsis | 90 | 1.19 | 6 | *S. suis* | MER, AMK, MTP | CSF PCR (+) *S. suis* | MER, AMK, MTP | NA | 5 |
| 47 | 44 yr/F | On admission | *S. suis* meningitis and sepsis | 9008 | 5.72 | 0.01 | *S. suis* | CRO, AMP, DEX | Blood culture (+) *S. suis* | CRO, DEX | NA | 5 |
| 48 | 61 yr/F | On admission | *S. suis* meningitis | 599 | 6.1 | 1.08 | *S. suis* | CRO, DEX | CSF PCR (+) *S. suis* | CRO, DEX | NA | 5 |
| 49 | 51 yr/M | On admission | *S. suis* meningitis and sepsis | 1116 | 1.56 | ND | *S. suis* | CRO, CIP, DEX | Blood culture (+) *S. suis* | CRO, CIP, DEX | NA | 5 |
| 50 | 59 yr/M | On admission | *S. suis* meningitis | 355 | 4.1 | 0.7 | Negative | CRO, TBM, DEX | CSF PCR (+) *S. suis* | CRO, TBM, DEX | NA | 5 |
| 51 | 82 yr/M | On admission | EBV meningoencephalitis | 40 | 1.27 | 3.1 | Negative | MER, AMK, DEX | CSF PCR (+) EBV | MER, AMK, DEX | NA | 3 |
| 52 | 57 yr/M | On admission | EBV meningitis | NA | 1.58 | 3.2 | Negative | MER, AMK, DEX | CSF PCR (+) EBV | MER, AMK, DEX | NA | 5 |
| 53 | 60 yr/M | On admission | EBV meningoencephalitis | 19 | 1.75 | 2.4 | Negative | CRO, CIP | CSF PCR (+) EBV | CRO, CIP, DEX | NA | 5 |
| 54 | 61 yr/M | On admission | *A. baumannii* meningitis and sepsis | 6903 | 21.96 | 0.01 | *A. baumannii* | MER, LNZ, DEX | None | MER, COL | NA | 2 |
| 55 | 78 yr/F | On admission | *T. asahii* meningitis, septic shock | 6 | 0.86 | 2.1 | *T. asahii* | MER, DEX | Urine culture (+) *T. asahii* | VOR, DEX | NA | 1 |
| 56 | 69 yr/M | On admission | *Streptococcus spp* meningitis | 5760 | 7 | 2.5 | Negative | CRO, CIP, DEX | CSF PCR (+) *Streptococcus spp* | CRO, LNZ, DEX | NA | 5 |
| 57 | 30 yr/F | On admission | HIV related central nervous system infection, suspected Cerebral toxoplasmosis | 3 | 0.45 | 1.9 | Negative | SMX, CLM, FLU, DEX | CSF PCR (+) HIV | SXT, FLU, MTP | NA | 3 |
| 58 | 35 yr/M | On admission | HSV meningoencephalitis | 14 | 0.83 | 6 | Negative | CRO, ACV, DEX | CSF PCR (+) HSV-1 | CRO, ACV, DEX | FN | 5 |
| 59 | 67 yr/M | During hospitalization | HSV encephalitis | 101 | 0.87 | 3.19 | Negative | MER, VAN ACV | CSF PCR (+) HSV-1 | ACV | FN | 2 |
| 60 | 39 yr/F | During hospitalization | HSV meningitis | 122 | 0.41 | 3.48 | Negative | CRO, ACV, DEX | CSF PCR (+) HSV-1 | ACV, DEX | FN | 5 |
| 61 | 92 yr/F | During hospitalization | Cryptococcal meningitis | 135 | 0.44 | 3.65 | Negative | CRO | CSF fungal culture (+) *C. neoformans* | AMB | FN | 5 |
| 62 | 45 yr/M | On admission | Cryptococcal meningitis | 117 | 2.37 | 0.34 | Negative | AMB, FLU RHZ, S, MOX | Fungal culture (CSF) (+) *C. neoformans* | AMB, FLU RHZ, S, MOX | FN | 2 |
| 63 | 62 yr/M | On admission | *E.coli* meningitis | 5157 | 8.3 | 0.02 | *E.coli* | MER, LNZ, LVF | None | MER, LNZ, LVF | FN | 2 |
| 64 | 75 yr/F | During hospitalization | VZV encephalitis | 2 | 0.49 | 3.1 | Negative | ACV | CSF PCR (+) VZV | ACV, DEX | FN | 3 |

**Abbreviations:** ACV, acyclovir; AMB, amphotericin B; AMK, amikacin; AMP, ampicillin; CFT, cefotaxime; CIP, ciprofloxacin; CNS, central nervous system; COL, colistin; CSF, cerebrospinal fluid; CRO, ceftriaxone; DEX, dexamethasone; EBV, Epstein–Barr virus; F, female; FAME, BioFire® FilmArray® Meningitis/Encephalitis Panel; FLU, fluconazole; FN; false negative; Glu, glucose (mmol/L); HSV, Herpes simplex virus; LNZ, linezolid; LVF, Levofloxacin; M, male; MER, meropenem; MOX, moxifloxacin; MTP, methylprednisolone; NA, not applicable; Pro, protein (g/L); RHZE, R = rifampin; H = isoniazid; Z = pyrazinamide; E = ethambutol; S, streptomycin; SXT, trimethoprim–sulfamethoxazole; TBM: tobramycin; VAN, vancomycin; VOR, voriconazole; VZV, Varicella zoster virus; WBC, white blood cells (cells/mm^3^).

^a^Outcome: 1 death; 2: vegetative state; 3: severe disability; 4: moderate disability; 5: mild or no disability.

**Supplementary Table S4. Empirical antibiotic regimen in patients with suspected bacterial meningitis according to Vietnam’s Ministry of Health.**

| **Patient group** | **Common bacterial pathogens** | **Standard therapy** | **Alternative therapies** |
| --- | --- | --- | --- |
| 0 to 4 weeks | *Enterobacteriaceae, Streptococcus agalactiae, Listeria monocytogenes.* | Cefotaxime plus Ampicillin | Ampicillin* plus Aminoglycoside** |
| 1 to 3 months | *Haemophilus influenzae b, Neisseria meningitidis, Streptococcus agalactiae E.coli, Listeria monocytogenes.* | Ampicillin* plus Ceftriaxone (or Cefotaxime) | Vancomycin plus Ceftriaxone (or Cefotaxime) |
| 3 months to 18 years | *Haemophilus influenzae b, Streptococcus pneumoniae, Neisseria meningitidis.* | Ceftriaxone (or Cefotaxime) | Vancomycin plus Ceftriaxone (or Cefotaxime) |
| Age > 18 and < 50 years | *Streptococcus pneumoniae, Streptococcus spp, Neisseria meningitidis.* | Ceftriaxone (or Cefotaxime) | Vancomycin plus Ceftriaxone (or Cefotaxime) |
| Age ≥ 50 years | *Streptococcus pneumoniae, Neisseria meningitidis, Listeria monocytogenes*, aerobic gram-negative bacilli. | Ceftriaxone (or Cefotaxime) | Ampicillin* plus Ceftriaxone (or Cefotaxime) |
| Immunodeficiency | *Streptococcus pneumoniae*, *Neisseria meningitidis*, *Listeria monocytogenes*, aerobic gram-negative bacilli. | Ampicillin plus Ceftazidime | Vancomycin plus Ampicillin* plus Ceftazidime |
| Head trauma, neurosurgery, CSF leak | *Streptococcus pneumoniae, Staphylococcus spp*, aerobic gram-negative bacilli. | Ceftazidime plus Vancomycin | Vancomycin plus Meropenem |

* Use of ampicillin in suspected cases of *Listeria monocytogenes*

**** Aminoglycoside (gentamycin or amikacin)
